# Carboxymethyl chitosan-folic acid-conjugated Fe_3_O_4_@SiO_2_ as a safe and targeting antitumor nanovehicle *in vitro*

**DOI:** 10.1186/1556-276X-9-146

**Published:** 2014-03-25

**Authors:** Hongmei Li, Zhen Li, Jin Zhao, Baoqiang Tang, Yanhong Chen, Yikun Hu, Zhengda He, Yue Wang

**Affiliations:** 1 State Key Laboratory of Coordination Chemistry, School of Chemistry and Chemical Engineering, Nanjing University, Nanjing 210008, China; 2 State Key Laboratory of Natural Medicines, School of Sciences, China Pharmaceutical University, Nanjing 211198, China

**Keywords:** Iron oxide, Surface functionalization, Tumor target, Surface modification

## Abstract

A synthetic method to prepare a core-shell-structured Fe_3_O_4_@SiO_2_ as a safe nanovehicle for tumor cell targeting has been developed. Superparamagnetic iron oxide is encapsulated inside nonporous silica as the core to provide magnetic targeting. Carboxymethyl chitosan-folic acid (OCMCS-FA) synthesized through coupling folic acid (FA) with OCMCS is then covalently linked to the silica shell and renders new and improved functions because of the original biocompatible properties of OCMCS and the targeting efficacy of FA. Cellular uptake of the nanovehicle was assayed by confocal laser scanning microscope using rhodamine B (RB) as a fluorescent marker in HeLa cells. The results show that the surface modification of the core-shell silica nanovehicle with OCMCS-FA enhances the internalization of nanovehicle to HeLa cells which over-express the folate receptor. The cell viability assay demonstrated that Fe_3_O_4_@SiO_2_-OCMCS-FA nanovehicle has low toxicity and can be used as an eligible candidate for drug delivery system. These unique advantages make the prepared core-shell nanovehicle promising for cancer-specific targeting and therapy.

## Background

Recently, considerable effort has been devoted to magnetic nanoparticles (NPs) as novel nanovehicles [1] and targeting agents [2] for biological and biomedical applications [3,4]. Iron oxide (Fe_3_O_4_) has emerged as one of the appealing candidates for drug delivery system [5] and magnetic fluorescence imaging [6,7]. However, the aggregations of naked Fe_3_O_4_ NPs decrease their interfacial areas, thus resulting in the loss of magnetism [8] and dispersibility [9]. Therefore, extensive work has been done to stabilize the NPs [10,11]. Huang synthesized uniform Fe_3_O_4_@SiO_2_ NPs with well-controlled shell thickness [12]. Kaskel developed a homogeneous Fe_3_O_4_@SiO_2_ with hollow mesoporous structure for drug delivery [13]. Unfortunately, the common challenge among these applications is to ensure sufficient uptake of NPs by specific cells [14,15]. The outer shell of silica not only protects the inner magnetite core from aggregation [16,17] but also provides sites for flexible surface modification such as poly(ethylene glycol) to render NP biocompatibility by preventing the nonspecific adsorption of proteins [18] and various targeting biomolecules [19,20] to improve the targeting efficiency. Kim reported Fe_3_O_4_@SiO_2_ NPs using CTAB as a template and PEG to prolong the short blood half-life of NPs [21]. However, the safety of drug carriers is one of the most critical factors to ensure its efficacy. Carboxymethyl chitosan (OCMCS) is a water-soluble chitosan which receives a great deal of interest because of favorable biocompatibility, safety, nonimmunogenicity, as well as reasonable cost [22]. Shi reported the OCMCS-Fe_3_O_4_ easily internalized into cells via endocytosis [23]. Fan developed the Fe_3_O_4_ NPs with OCMCS which significantly reduced the cytotoxicity and the capture of NPs. Moreover, folic acid (FA)-modified OCMCS-Fe_3_O_4_ NPs combined receptor-mediated targeting and magnetic targeting together [24]. It is noted that folic acid, as an effective target ligand [25,26], shows high binding affinity with folate receptor, which over-expressed on the membranes of many human malignant cells, but limited on the normal cells. To the best of our knowledge, the general synthetic protocols to combine silica with diverse functional modification used as a safe drug delivery system are seldom reported. With regard to the above effects, we develop a novel carboxymethyl chitosan-based, silica-coated iron oxide nanovehicle (Fe_3_O_4_@SiO_2_-OCMCS-FA) with dual-targeting function (magnetic/folate) in this study. Fe_3_O_4_ core serves as a carrier for magnetic targeting, while silica coating on the iron oxide NPs offers sites for further modifications. OCMCS-FA was conjugated firstly to perform a folate receptor (FR)-mediated cellular endocytose and acted as the biocompatible segment and then subsequently coupled through acylation to the surface of animated Fe_3_O_4_@SiO_2_ which was modified with (3-aminopropyl) triethoxysilane (APTES) to obtain the multifunctional nanovehicle (Fe_3_O_4_@SiO_2_-OCMCS-FA). Its uptake by human cervical carcinoma cell lines (HeLa cells) is traced, and the cytotoxicity on the human tumor cells and normal cells are both evaluated. The results show that it is nontoxic to them, which reveal that it could be used as a promising candidate for drug target delivery system.

## Methods

### Reagent materials

All chemicals are analytical reagent grade and were used as received. Folic acid is a biological reagent purchased from Sinopharm Chemical Reagent Co., Ltd., Shanghai, China.

### Synthesis of magnetic Fe_3_O_4_@SiO_2_ NPs

**Figure 1:**
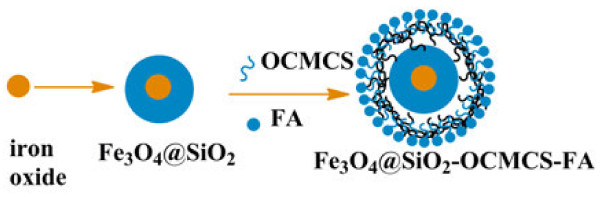
**Synthesis of Fe**_**3**_**O**_**4**_**@SiO**_**2**_**-OCMCS-FA.**

Monodispersed Fe_3_O_4_ NPs were prepared by the thermal decomposition of ferric acetylacetonate precursor in the presence of an oleic acid stabilizer and oleylamine [27]. SiO_2_ coating on the Fe_3_O_4_ NPs was performed through the formation of water-in-cyclohexane reverse microemulsion [28] (Figure 1).

Polyoxyethylene(5) nonylphenyl ether (5 mL, Igepal CO-520, Sigma-Aldrich, St. Louis, MO, USA) was firstly dispersed in cyclohexane (40 mL). Then, 2 mL Fe_3_O_4_ solution (50 mg mL^-1^ in cyclohexane) was added. After 10 min, ammonium hydroxide (292 μL) was added to form a transparent brown solution of reverse microemulsion. Next, tetraethylorthosilicate (TEOS) was added and the reaction was continued at room temperature for 24 h. When isopropanol was added into the reaction solution, Fe_3_O_4_@SiO_2_ NPs were precipitated. They were collected by centrifugation and washed with ethanol. Fe_3_O_4_@SiO_2_ NPs were then dried in vacuum at 60°C.

### Synthesis of OCMCS-FA conjugate

**Figure 2:**
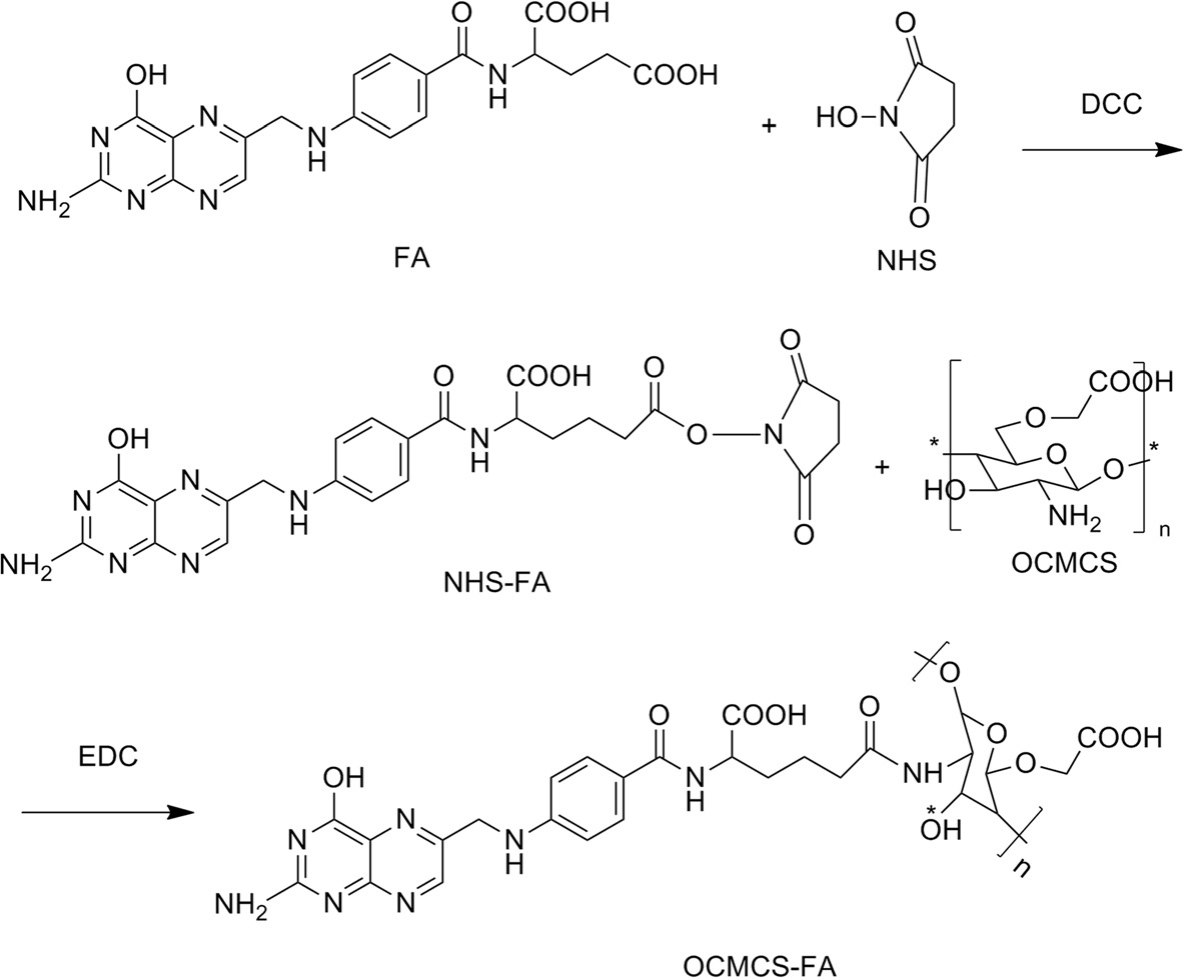
Synthesis of OCMCS-FA.

The synthesis of OCMCS-FA conjugate was adopted by homogeneous synthesis through acylation (Figure 2). Folic acid (0.884 g) was dissolved in 20 mL of anhydrous dimethylsulfoxide (DMSO) to which dicyclohexylcarbodiimide (DCC; 0.784 g) and *N*-hydroxysuccinimide (NHS; 0.256 g) were added. The reaction mixture was stirred for 24 h at 45°C in the dark [29]. The by-product dicyclohexylurea was filtered off, and 20 mL of 30% acetone in diethyl ether was added with stirring. A yellow precipitate (NHS-FA) formed and was collected after washing with diethyl ether several times. Then, 100 mg OCMCS was dissolved in acetate buffer (pH 4.7). A mixture solution of NHS-FA and 1-ethyl-3-(3-dimethylaminopropyl) carbodiimide (EDC) was prepared by dissolving NHS-FA and EDC simultaneously in DMSO. Finally, the mixture solution was dropped into the OCMCS solution. After 24 h, the solution was adjusted to pH 9 with NaOH and purified by centrifugation followed by 2 days of dialysis against phosphate-buffered solution (PBS) and extensive dialysis against water using a 3,500-Da cutoff dialysis membrane. OCMCS-FA was then dried in vacuum at 60°C.

### Synthesis of Fe_3_O_4_@SiO_2_-OCMCS-FA NPs

APTES was anchored to the surface of Fe_3_O_4_@SiO_2_ through refluxing at 110°C in toluene to develop amide in the surface of silica in order to introduce carboxyl groups of OCMCS-FA conjugate. Fifty milligrams of APTES-modified Fe_3_O_4_ was added to 10 mL of a 2-(*N*-morpholino) ethanesulfonic acid buffer (0.1 M, pH 6.5) containing 50 mg of OCMCS-FA, EDC (20 mM), and NHS (50 mM). The mixture suspension was then sonicated for 10 min in ultrasonic disrupter and shaken for 24 h at room temperature. The OCMCS-FA bound Fe_3_O_4_@SiO_2_ were collected under centrifugation, washed with ethanol, and dried in vacuum at 60°C.

### Hemolysis assay

Two milliliters of rat blood was injected from the eye socket vein. The red blood cells (RBCs) were obtained by removing the serum from the blood by centrifugation and suction. After being washed with PBS solution five times, the RBCs were diluted to 1/10 of their volume with PBS solution. Diluted RBC suspension (0.3 mL) was then mixed with the following: (a) PBS (1.2 mL) as a negative control, (b) deionized water (1.2 mL) as a positive control, and (c) nanovehicle suspensions (1.2 mL) at concentrations ranging from 40 to 500 μg mL^-1^. The mixtures were then vortexed and kept for 2 h at room temperature. Finally, the mixtures were centrifuged for 2 min at 4,000 rpm and the absorbance of the upper supernatants at 541 nm was measured by UV-visible (UV-vis) characterization. The percentage of hemolysis was calculated using the following equation (*A* is the absorbance of UV-vis spectra) [30]:

$$\text{Hemolysis}=\left(A_{\text{sample}}-A_{(-)\text{control}}\right)/\left(A_{(+)\text{control}}-A_{(-)\text{control}}\right)$$

### Cell culture and uptake

HeLa cell lines were maintained in Dulbecco's modified Eagle's medium (DMEM) containing 10% fetal bovine serum, 100 units mL^-1^ penicillin, and 100 mg mL^-1^ streptomycin in 37°C, 5% CO_2_. For investigation on targeting of nanovehicles, nanovehicles were labeled with RB to form RBFe_3_O_4_@SiO_2_ and RBFe_3_O_4_@SiO_2_-OCMCS-FA nanoparticles [31]. In a typical procedure, 2.5 × 10^4^ cells were seeded in a 35-mm dish with a glass bottom for 24 h to allow the cells to attach. After the cells were washed twice with PBS, the samples were added to the dishes in a concentration of 100 μg mL^-1^. After 2 h of incubation, the cells were washed several times with PBS to remove the remaining samples and dead cells. Finally, the cells were observed under a confocal laser scanning microscope (CLSM; Carl Zeiss LSM 710, Oberkochen, Germany). Cells with the addition of Fe_3_O_4_@SiO_2_ were imaged as control.

### Bio-TEM observations for HeLa cells

The HeLa cells were incubated with 2.5 μg mL^-1^ nanovehicle inDMEM in 5% CO_2_ at 37°C for 24 h. Afterwards, cells were washed three times with PBS and subsequently fixed with 2.5% glutaraldehyde in 0.03 M potassium phosphate buffer for at least 24 h. Cells were then washed in PBS, postfixed with 1% osmium tetroxide in sodium carboxylate buffer, washed with 0.05 mol L^-1^ maleate, and stained with 0.5% uranylacetate (Sigma-Aldrich) in maleate buffer. After washing the cells in 0.05 mol L^-1^ maleate, the cells were dehydrated in a grading series of ethanol followed by acetone, embedded in Epon (Momentive Specialty Chemicals, Inc., Columbus, OH, USA), and dried in an oven at 60°C for 4 days. Ultrathin sections of approximately 50 nm thick were cut with a diamond knife on a Leica Ultracut R ultramicrotome (Milton Keynes, UK) and transferred to the copper grid. The images were viewed on JEOL-2100 electron microscope (Akishima, Tokyo, Japan).

### Cytotoxicity

The *in vitro* cytotoxicity was measured by using the 3-(4,5-dimethylthiazol-2-yl)-2,5-diphenyltetrazolium bromide (MTT) assay in HeLa cells. Cells were initially seeded into a 96-well cell culture plate at 1 × 10^4^ per well and then incubated for 24 h at 37°C under 5% CO_2_. DEME solutions of nanovehicle at concentrations of 100 mg mL^-1^ were added to the wells. The cells were further incubated for 72 h at 37°C under 5% CO_2_. The cells were washed three times with 0.2 mL PBS to remove the unbound nanoparticles. Subsequently, 0.2 mL DEME and 25 mL MTT (5 mg mL^-1^) were added to each well and incubated for an additional 4 h at 37°C under 5% CO_2_. Then, the medium solution was replaced by 0.15 mL DMSO solution. After 10 min, the optical density at 490 nm (absorption value) of each well was measured on a Tecan Infinite M 200 monochromator-based multifunction microplate reader (Männedorf, Switzerland). The corresponding nanovehicle with cells but not treated by MTT were used as controls. The cell vitality after labeling was compared with that of unlabeled cells and expressed as the relative ratio.

### Characterization

^1^H NMR spectra was recorded at 300 MHz on a Bruker ARX 300 spectrometer (Ettlingen, Germany). Infrared spectra (4,000 to 400 cm^-1^) were recorded on Bruker Fourier transform infrared (FTIR) spectrometer in KBr pellets. The X-ray powder diffraction patterns were recorded on an X'Pert diffractometer (PANalytical B.V., Almelo, The Netherlands) with CuKα radiation (*λ* = 1.54060 Å) at 45 kV and 40 mA. X-ray photoelectron spectroscopy (XPS) analysis was performed with a ESCALB MK-II (Physical Electronics Instruments, Chanhassen, MN, USA). The source was the monochromatic MgKα radiation. The surface charge of the nanovehicles was investigated on Malvern Zetasizer Nano ZS 90 zeta potential analyzer (Westborough, MA, USA). Transmission electron microscopy (TEM) was performed on a JEOL-2100 with an accelerating voltage of 200 kV. TEM samples were prepared by drop-casting dispersion onto copper grids covered by carbon film. Ultrathin sections for bio-TEM were cut with a diamond knife on a Leica Ultracut R ultramicrotome. Scanning electron microscopy (SEM) was performed on a JEOL-S-3400 N II. Magnetic property measurements were performed using a Quantum Design MPMS XL-7 superconducting quantum interference device (SQUID; Olomouc, Czech Republic). Confocal images were acquired using a Zeiss confocal laser scanning unit mounted on an LSM 710 fixed-stage upright microscope.

## Results and discussion

**Figure 3:**
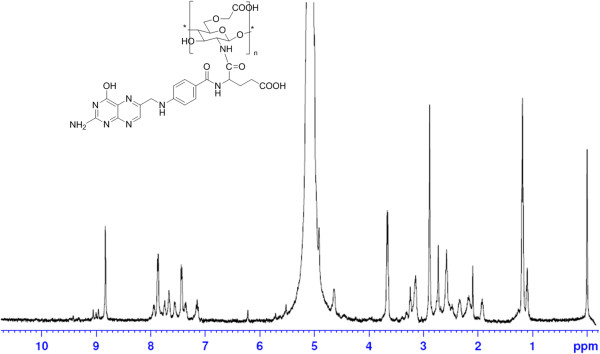
^**1**^**H NMR spectra of OCMCS-FA in CF**_**3**_**COOD/D**_**2**_**O.**

The ^1^H NMR spectra of OCMCS-FA conjugate was shown in Figure 3. The signals at *δ* 1.65, 2.88, and 3.08 to 3.64 ppm was assigned to the resonance of the monosaccharide residue protons, -COCH_3_, -CH-NH-, and -CH_2_-O-, respectively. The signals appearing at *δ* 6.3 to 8.5 ppm were attributed to the resonance of the folate aromatic protons. So, it revealed that the couple of the FA residue to the OCMCS could be achieved via EDC mediation [32].

**Figure 4:**
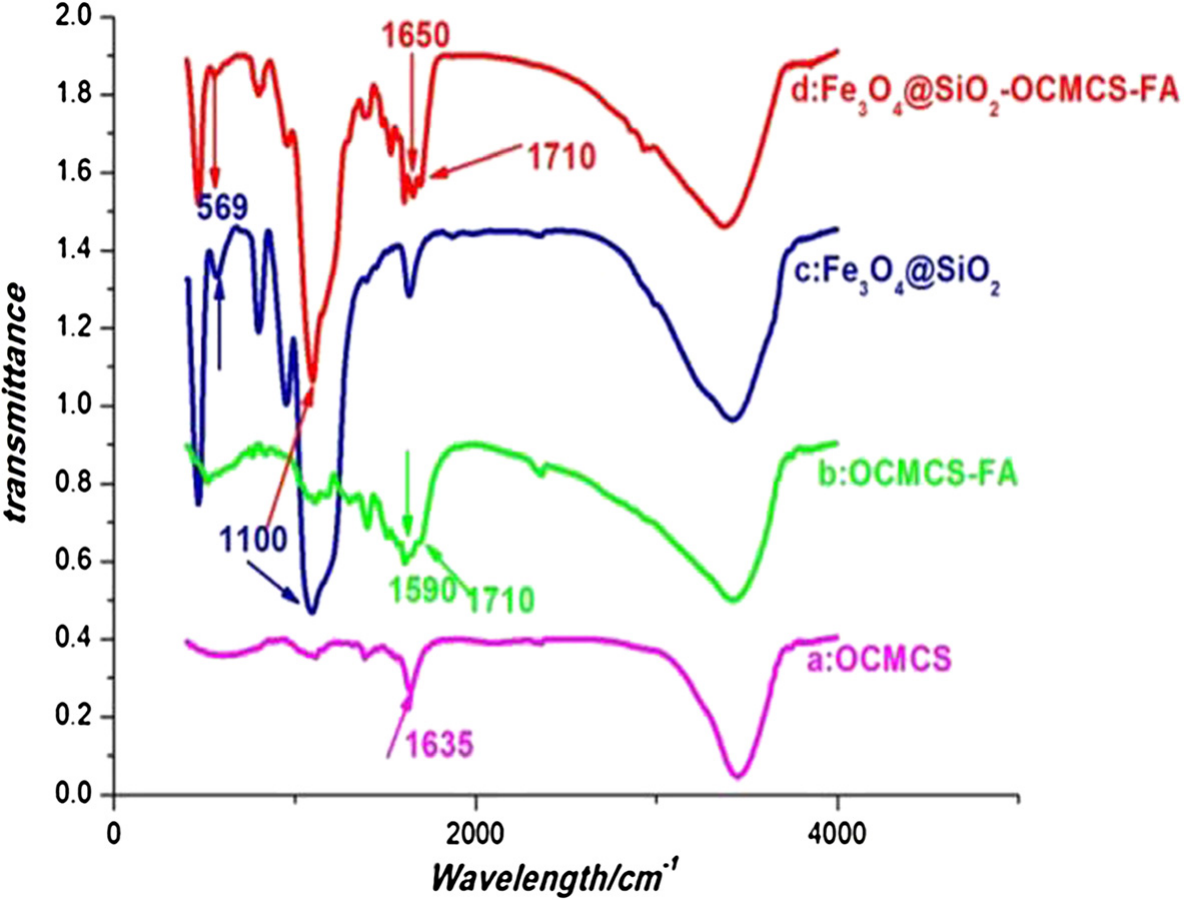
**FTIR spectra. (a)** OCMCS, **(b)** OCMCS-FA, **(c)** Fe_3_O_4_@SiO_2_, and **(d)** Fe_3_O_4_@SiO_2_-OCMCS-FA.

FTIR spectroscopy shown in Figure 4 confirmed that OCMCS-FA was successfully immobilized on the Fe_3_O_4_@SiO_2_ NPs. In the spectrum of OCMCS-FA (Figure 4b), the 1,635 cm^-1^ peak of COO- stretching vibration shifted to 1,590 cm^-1^ compared to OCMCS (Figure 4a). Moreover, a shoulder peak around 1,710 cm^-1^ is observed in OCMCS-FA which verified that FA conjugated to the OCMCS successfully [33]. The bare Fe_3_O_4_ NPs showed characteristic bands related to the Fe-O vibrations near 569 cm^-1^ (Figure 4b,c). The peak at 1,100 cm^-1^ indicated Si-O bonding on the NP surface (Figure 4c). Unsurprisingly, the FTIR spectra for Fe_3_O_4_@SiO_2_-OCMCS-FA nanovehicle presented similar peaks at 1,710, 1,590, 1,100, and 569 cm^-1^ (Figure 4d). What is more, the FTIR spectrum of Fe_3_O_4_@SiO_2_-OCMCS-FA nanovehicle displayed an intense peak at 1,650 cm^-1^ which might result from the -CONH- due to the reaction between the carboxyl group of the OCMCS and amide on the surface of silica.

**Figure 5:**
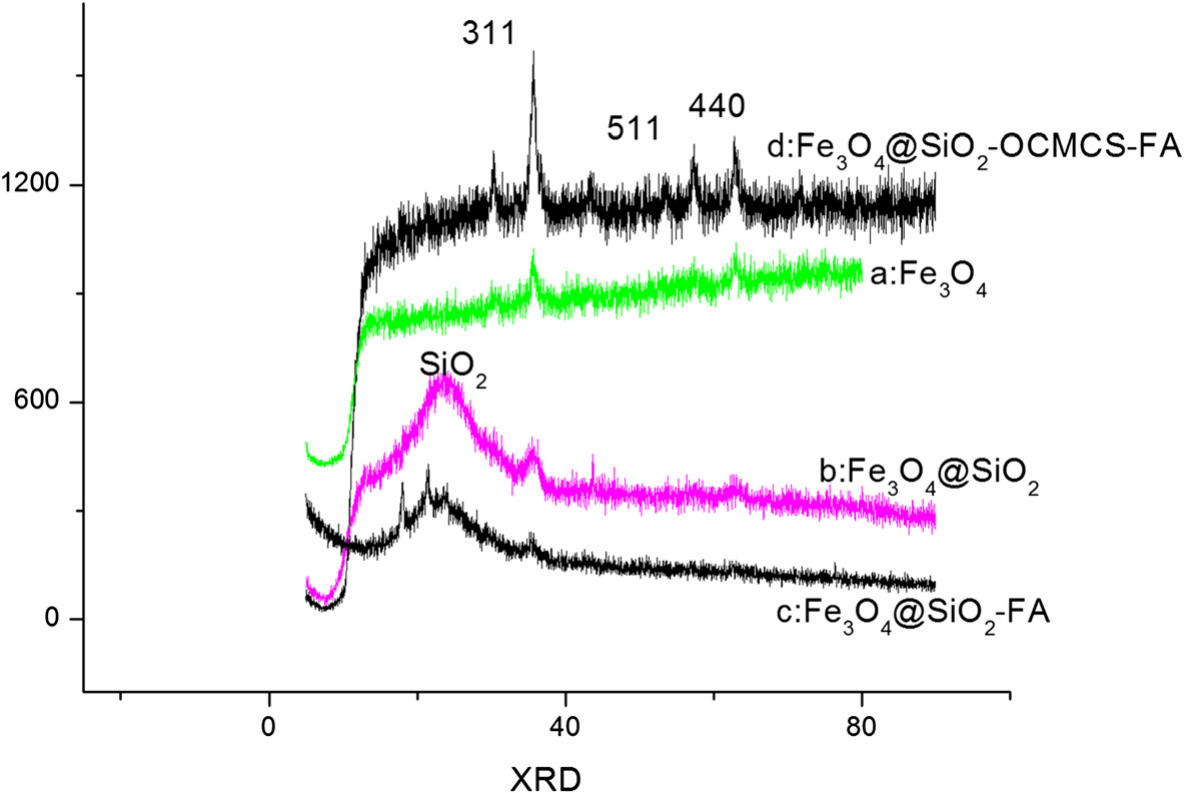
**XRD spectrum. (a)** Fe_3_O_4_ NPs, **(b)** Fe_3_O_4_@SiO_2_, **(c)** Fe_3_O_4_@SiO_2_-FA, and **(d)** Fe_3_O_4_@SiO_2_-OCMCS-FA.

The XRD measurements were performed with the dried powder samples of bare, silica-coated and OCMCS-FA-conjugated iron oxide to identify the crystal phases. The pattern of OCMCS-FA-conjugated NPs (Figure 5) showed all the major peaks corresponding to Fe_3_O_4_ which could be assigned to the (311), (511), and (440) planes, respectively [34]. Additionally, the peak around 2*θ* = 25° due to the silica [35] was observed in the case of the silica-coated NPs, but disappeared in the Fe_3_O_4_@SiO_2_-OCMCS-FA nanovehicle which may attribute to the OCMCS-FA conjugated. These results confirmed the surface modification of the Fe_3_O_4_ NPs with OCMCS-FA.

**Figure 6:**
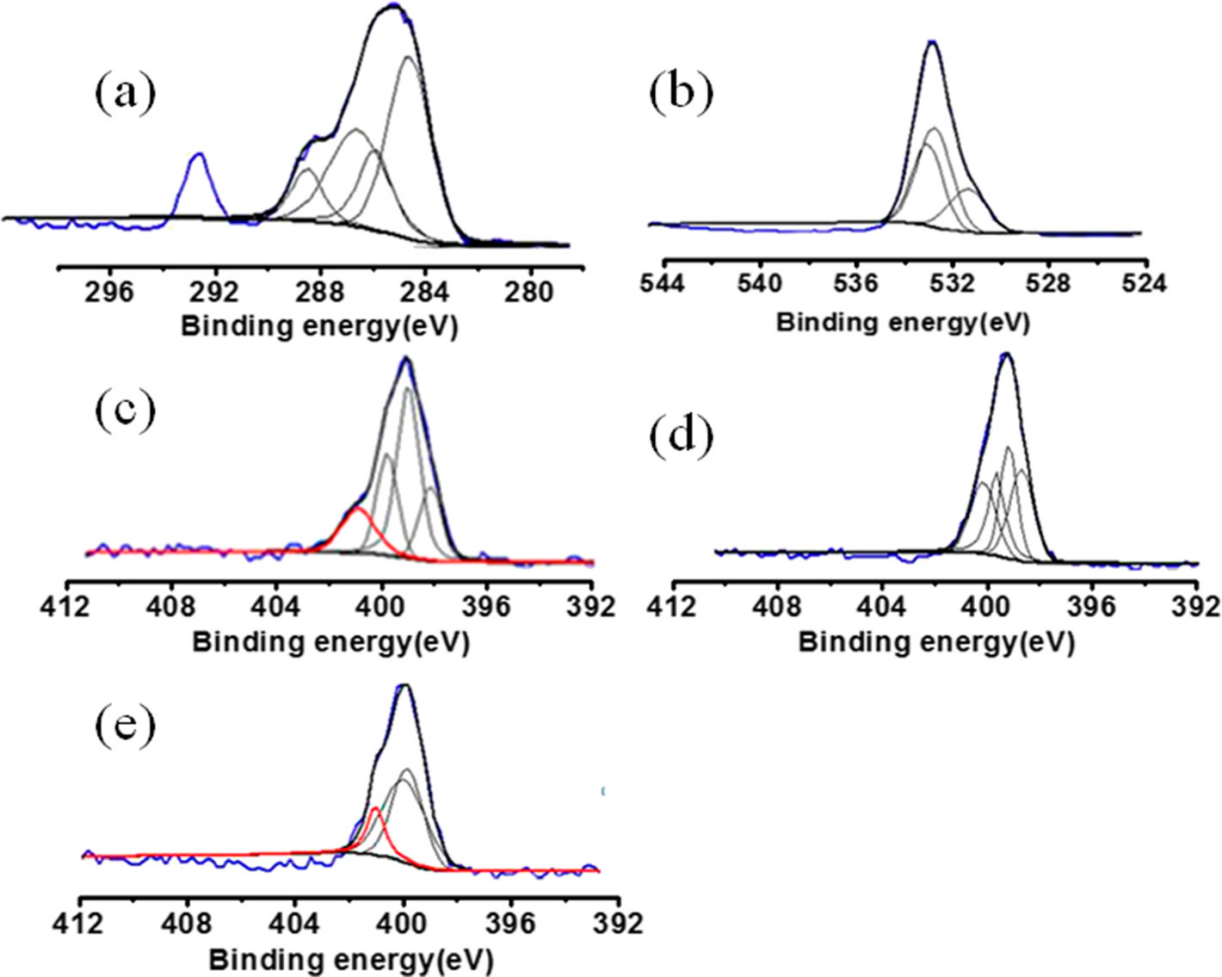
**High-resolution C 1s, O 1s, and N 1s X-ray photoelectron spectra. (a)** High-resolution C 1s spectrum of Fe_3_O_4_@SiO_2_-OCMCS-FA, **(b)** high-resolution O 1s spectrum of Fe_3_O_4_@SiO_2_-OCMCS-FA, **(c)** high-resolution N 1s spectrum of Fe_3_O_4_@SiO_2_-OCMCS-FA, **(d)** high-resolution N 1s of OCMCS-FA, and **(e)** high-resolution N 1s spectrum of FA.

The surface composition was also ascertained by XPS as it is recognized as a quantitative surface elemental analysis and chemical state information. Wide-scan spectra were acquired for NPs with high-resolution C 1s, O 1s, and N 1s. Spectral calibration was carried out by setting the main C 1s peak at 285 eV. The high-resolution scans for C 1s (Figure 6a) of Fe_3_O_4_@SiO_2_-OCMCS-FA nanovehicle could be deconvoluted into four peaks at 285.7, 284.5, 286.3, and 288.2 eV, which could be attributed to -C-O-, -C-C-, -NH-C = O, and -COOH groups, respectively. The O 1 s spectrum (Figure 6b) of nanovehicle displayed three peaks at 532.3, 532.6, and 530.9 eV corresponding to oxygen being present in three different environments as -C-O, -O-H, and C = O in Fe_3_O_4_@SiO_2_-OCMCS-FA nanovehicle. Compared with the free folate, OCMCS-FA, and Fe_3_O_4_@SiO_2_-OCMCS-FA, distinction was made towards the high-resolution scans for N 1s. Free folate (Figure 6e) could be deconvoluted into four peaks at 399. 9, 400.1, 399.5, and 398.5 eV. The bands at 398.5 and 399.5 eV are due to the amide N and other N of FA, respectively. The bands at 400.1 and 399.9 eV were in accordance with those of triazole ring N as reported [36]. However, the peak of free amide N at 398.5 eV disappeared in the spectrum of OCMCS-FA (Figure 6d), and a new peak at 400.8 eV appeared due to the amide conjugation between FA and OCMCS. Interestingly, the N 1-s spectrum of Fe_3_O_4_@SiO_2_-OCMCS-FA nanovehicle (Figure 6c) showed similar peaks with OCMCS-FA except at 401.2 eV. The peak at 401.2 eV might be originated from the formation of amide linkage between the carboxyl group of the OCMCS and amide on the surface of silica which was reasonably consistent with the peak reported in the literature. Anyway, XPS results support OCMCS-FA chemically bound to the surface of Fe_3_O_4_@SiO_2_ by amidation.

**Figure 7:**
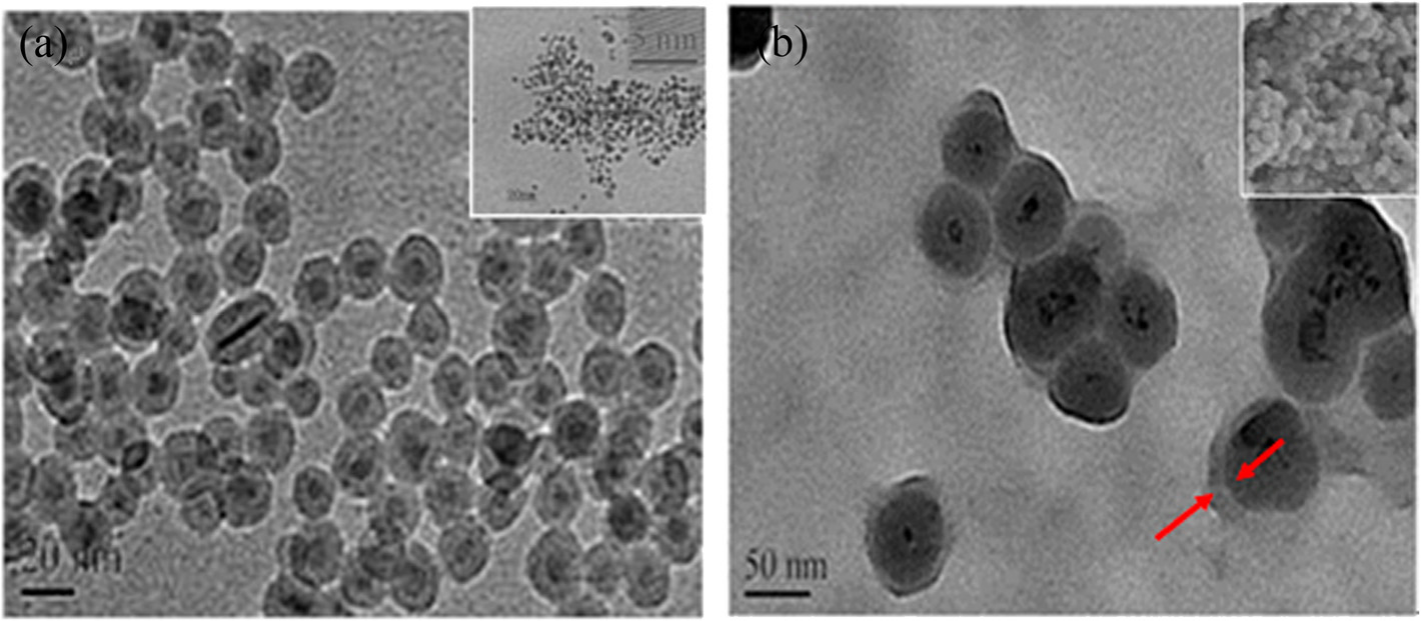
**TEM images. (a)** Fe_3_O_4_@SiO_2_ (inset: Fe_3_O_4_) and **(b)** Fe_3_O_4_@SiO_2_-OCMCS-FA (inset: SEM images of Fe_3_O_4_@SiO_2_-OCMCS-FA).

Moreover, the zeta potential of suspension for Fe_3_O_4_@SiO_2_-OCMCS-FA was -28.89 ± 0.43 mV which was smaller than that of Fe_3_O_4_ NPs considering that silica and OCMCS-FA modification protect the Fe_3_O_4_ NPs away from aggregation. As shown in Figure 7, spherical Fe_3_O_4_ NPs were chosen as the template to obtain multifunctional nanovehicle. It can be seen that spherical Fe_3_O_4_ NPs were about 6 to 8 nm in size with high dispersibility (Figure 7a, inset). The corresponding high-resolution image (Figure 7a, inset) showed clear lattice fringes which corresponds to Fe_3_O_4_. A thick layer of dense silica was deposited onto the surface of Fe_3_O_4_ with a core thickness of 7 nm and shell thickness of 14 nm (Figure 7a) with uniform particle size and excellent morphology. Then, a thin layer of OCMCS-FA conjugated to the surface of Fe_3_O_4_@SiO_2_ through amidation with the aid of sodium tripolyphosphate (TPP) forms a tri-layered (5 nm) multifunctional nanovehicle (Fe_3_O_4_@SiO_2_-OCMCS-FA) (Figure 7b). The SEM image shows that the nanovehicles are very uniform in both size and shape (Figure 7b, inset).

**Figure 8:**
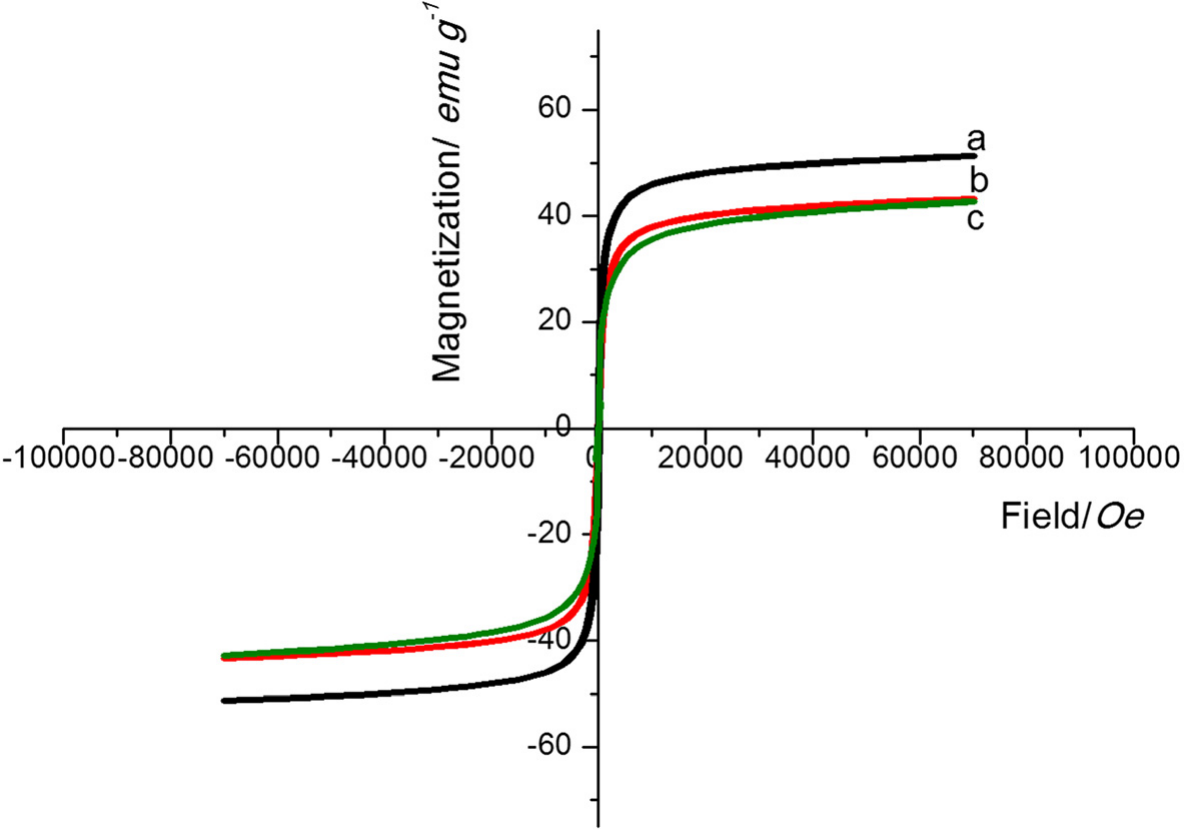
**Magnetization curve. (a)** Fe_3_O_4_**(b)** Fe_3_O_4_@SiO_2_, and **(c)** Fe_3_O_4_@SiO_2_-OCMCS-FA nanovehicle at 300 K.

The magnified hysteresis loop of Fe_3_O_4_@SiO_2_-OCMCS-FA nanovehicle which clearly showed that no remanence and hysteresis were detected demonstrated the superparamagnetism of the nanovehicle (Figure 8). After coating with silica, the magnetization of Fe_3_O_4_@SiO_2_ was undoubtedly decreased compared with the Fe_3_O_4_ nanoparticles for the shell and relatively low Fe_3_O_4_ amount. However, after the final modification of OCMCS-FA, the magnetization of the nanovesicles was not apparently decreased due to the thin outer layer. Factually, superparamagnetism is usually highly desired because it can prevent the magnetic composite particles from irreversible aggregation and ensure an excellent dispersibility once the applied magnetic field is removed [37].

### *In vitro* targeting of nanovehicle

**Figure 9:**
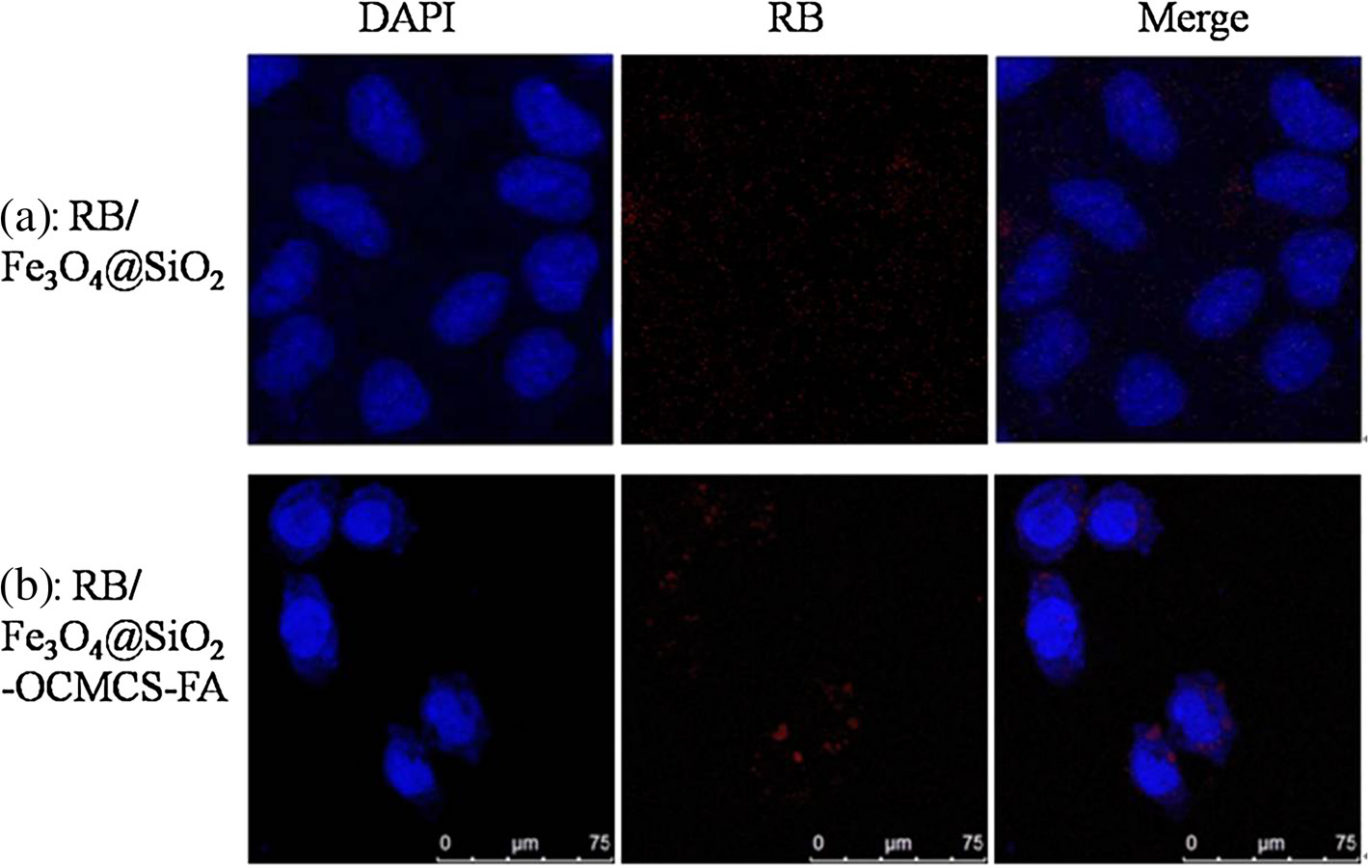
**Confocal laser scanning microscope images of subcellular localization. (a)** RBFe_3_O_4_@SiO_2_ and **(b)** RBFe_3_O_4_@SiO_2_-OCMCS-FA after 2 h of incubation with HeLa cells. Nuclei were stained with DAPI.

The ability of nanoparticles to target specific locations is one of the most important factors for their prospective application in drug delivery and biomedicine. To investigate the uptake possibility of Fe_3_O_4_@SiO_2_-OCMCS-FA, CLSM was applied to trace the process of this nanovehicle. Therefore, RB is labeled on the surface of the nanovehicle to distinguish it. To explore the practical application of this nanovehicle in the targeting of tumor cells, the particles were incubated in physiological conditions with HeLa cells bearing the over-expressed folate receptor. Figure 9 shows DAPI, RB, and merged images of HeLa cells incubated with RBFe_3_O_4_@SiO_2_ (20 μg mL^-1^, control) and RBFe_3_O_4_@SiO_2_-OCMCS-FA (20 μg mL^-1^) for 2 h. Interestingly, even at the very low concentration, the CLSM images show that the RBFe_3_O_4_@SiO_2_-OCMCS-FA nanoparticles could be taken up by HeLa cells within a short period as manifested by the appearance of spot-like red fluorescence in cells (Figure 9b), while untreated RBFe_3_O_4_@SiO_2_ showed negligible background fluorescence under similar imaging conditions (Figure 9a). The merge of the bright-field and fluorescent images further demonstrates that the luminescence is strongly correlated with the intracellular location (Figure 9b) suggesting the feasibility and efficiency of the nanoparticles for anticancer drug delivery into cancer cells. In addition, the fluorescent image shown in Figure 9b also testifies that the nanovehicle was mainly distributed in the cytoplasm after cellular uptake. The confocal laser scanning microscope observation confirms that the nanovehicle could be effectively taken up by the HeLa cells as the folate modified.

**Figure 10:**
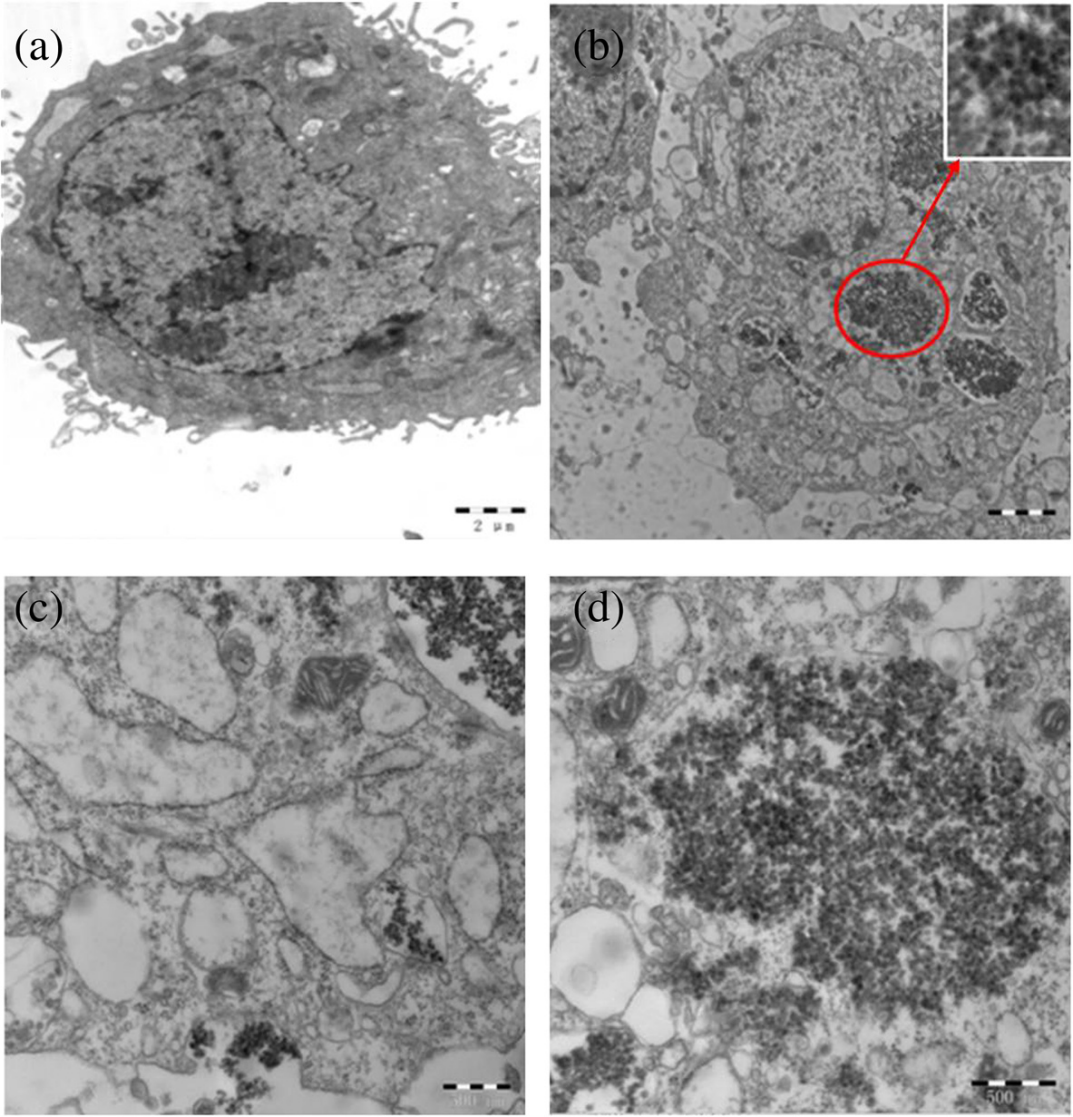
**Bio-TEM images of HeLa cells after 24 h of exposure to NPs (100 μg mL**^**-1**^**). (a)** Control, **(b)** Fe_3_O_4_@SiO_2_-OCMCS-FA nanovehicle (inset: magnified image of the circled area) and **(c, d)** magnified image of Fe_3_O_4_@SiO_2_-OCMCS-FA nanovehicle.

To further reveal that the nanovehicle was internalized in HeLa cells rather than being bound to the cell surface, bio-TEM was used to analyze the nanovehicle-treated cells. Unlike the untreated cells (Figure 10a), some aggregates of nanovehicles were observed as black patches inside the cell cytoplasm which maintained their core-shell structure (Figure 10b and the inset), while no nanovehicle was found in the nucleus which coincided with the results of CLSM. Based on the cell morphology, it is plausible that the nanovehicle accumulates on the membrane (Figure 10c) by the high specific interaction between folic acid on the nanovehicle and FR on HeLa cells which may increase the uptake through folate receptor-mediated endocytosis. Afterwards, majority of the internalized nanovehicle will be processed in lysosomes and are eventually released into the cytoplasm (Figure 10d). Therefore, *in vitro* CLSM and bio-TEM images present evidence about the target effects of nanovehicle with the OCMCS-FA modification.

### Biocompatibility of nanovehicles (hemolysis assay and cytotoxicity)

**Figure 11:**
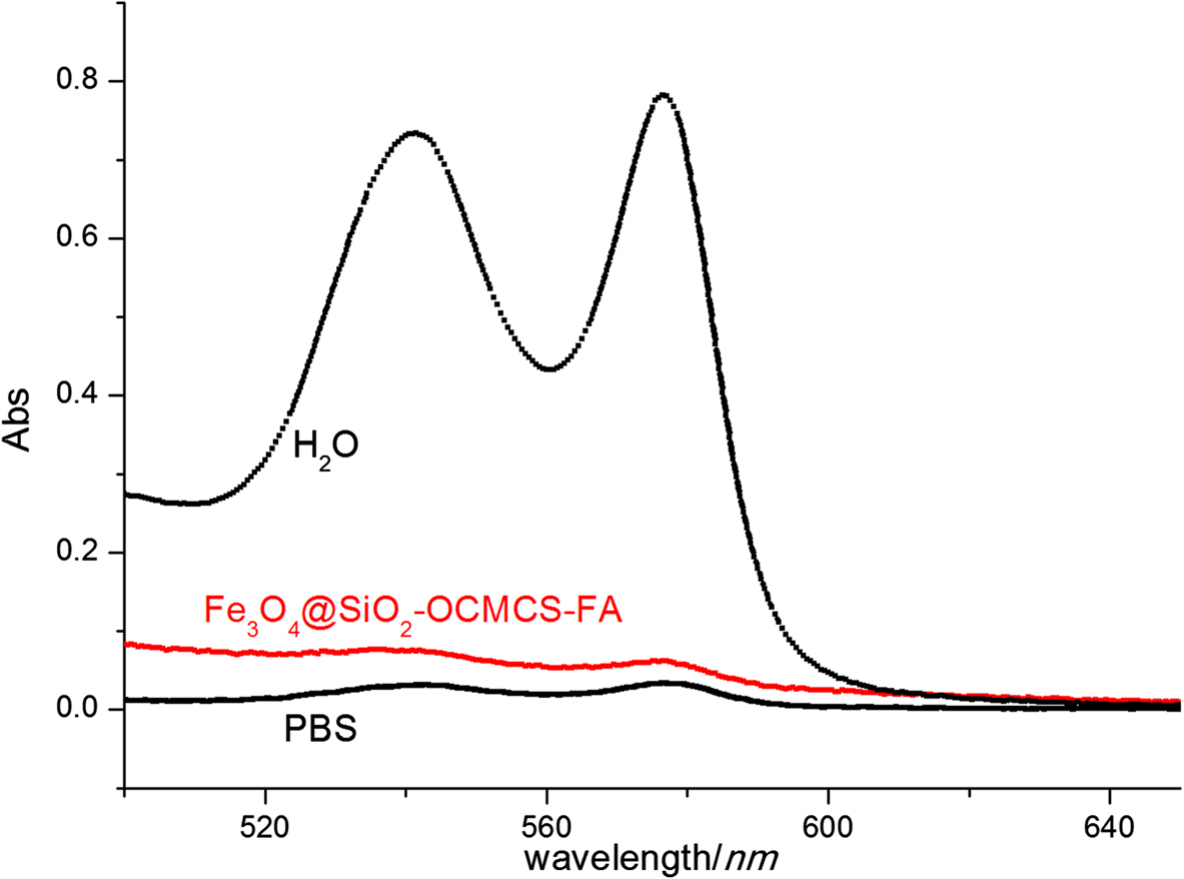
**Percentage of hemolysis of RBCs in the presence of Fe**_**3**_**O**_**4**_**@SiO**_**2**_**-OCMCS-FA at 500 μg mL**^**-1**^**.** Water (+) and PBS (-) are used as positive and negative controls, respectively.

It is important to investigate the biocompatibility of Fe_3_O_4_@SiO_2_-OCMCS-FA nanovehicles when materials are administrated by vein injection. Hemolysis assay is a primary approach to assess the biocompatibility for *in vivo* applications. The hemolysis percentage of the nanovehicles was quantified based on the absorbance of the supernatant at 541 nm with isotonic PBS and distilled water as control. From Figure 11, Fe_3_O_4_@SiO_2_-OCMCS-FA nanovehicle exhibits good biocompatibility, and the hemolysis percentage of Fe_3_O_4_@SiO_2_-OCMCS-FA even at a high concentration of 500 μg mL^-1^ was 6.3% lower than the value of traditional nanoparticles (70% of 500 μg mL^-1^) [38]. Thus, the obtained results showed that no visible hemolysis effect was observed visually for nanovehicle to evidence the good blood compatibility for the introduction of OCMCS.

**Figure 12:**
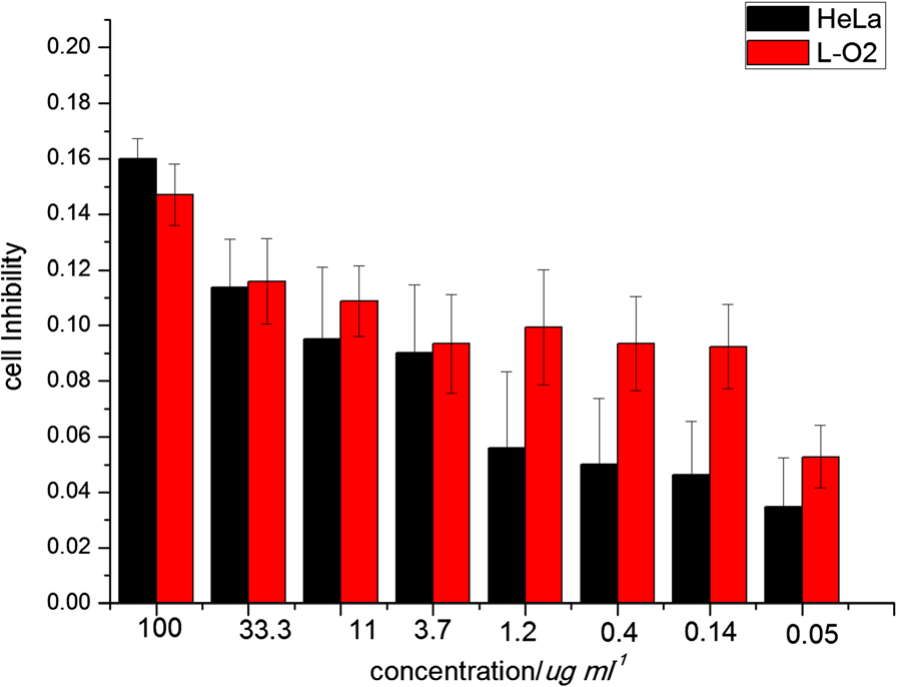
**Cell inhibition of Fe**_**3**_**O**_**4**_**@SiO**_**2**_**-OCMCS-FA nanovehicle on HeLa and L-O2 cells.**

In order to verify the toxicity of nanovehicle, *in vitro* cytotoxicity of the nanovehicle on HeLa and human liver cells (L-O2) was evaluated using a traditional MTT assay. The results (Figure 12) showed that there was a relatively high cell viability (more than 80% at a concentration of 100 μg mL^-1^) in HeLa which displays low cytotoxicity and favorable cell compatibility which is consistent with hemolysis assay. In addition, the viability of the L-O2 cells was similar to that of the HeLa after incubating with nanovehicle which demonstrates that Fe_3_O_4_@SiO_2_-OCMCS-FA possesses safety for normal cells as a drug carrier. The mesoporous silica layer of this nanovehicle is currently studied by our group, which may offer the platform for insoluble drugs in biomedical application.

## Conclusions

In summary, we presented a rational method of preparing folic acid-conjugated carboxymethyl chitosan by homogeneous synthesis characterized by ^1^H NMR and FTIR. Moreover, a novel, safe, and tumor-targeting nanovehicle with iron oxide as core and silica as shell has been fabricated showing good dispersion. It was firstly reported that OCMCS-FA conjugated on the surface of Fe_3_O_4_@SiO_2_ via amide reaction to form the layer of compatibility and receptor-mediated targeting. Fe_3_O_4_@SiO_2_-OCMCS-FA nanovehicle exhibits high uptake of HeLa cells which imply low cytotoxicity and good biocompatibility because of the dual targeting and OCMCS serving as a long circulation. Furthermore, the silica moiety of Fe_3_O_4_@SiO_2_-OCMCS-FA nanovehicle could be extended to fabricate mesoporous nanovehicle which may increase surface area and pore volume. Thus, we believe that this strategy may provide a safe and efficient platform for antitumor drug delivery.

## Competing interests

The authors declare that they have no competing interests.

## Authors’ contributions

HL and YW conceived and designed the experimental strategy and wrote the manuscript. JZ and YC prepared andperformed the synthetic experiments. YH analyzed the data. ZH and BT performed the *in vitro* experiments. ZL helped with the editing of the paper. All authors read and approved the final manuscript.
